# Efficient Generation of Hydrogen Peroxide and Formate by an Organic Polymer Dots Photocatalyst in Alkaline Conditions

**DOI:** 10.1002/anie.202202733

**Published:** 2022-04-05

**Authors:** Sicong Wang, Bin Cai, Haining Tian

**Affiliations:** ^1^ Department of Chemistry—Ångström Laboratory Uppsala University 751 20 Uppsala Sweden

**Keywords:** Alkaline Condition, Formate, Hydrogen Peroxide, Photocatalysis, Polymer Dots

## Abstract

A photocatalyst comprising binary organic polymer dots (Pdots) was prepared. The Pdots were constructed from poly(9,9‐dioctylfluorene‐*alt*‐benzothiadiazole), as an electron donor, and 1‐[3‐(methoxycarbonyl)propyl]‐1‐phenyl‐[6.6]C_61_, as an electron acceptor. The photocatalyst produces H_2_O_2_ in alkaline conditions (1 M KOH) with a production rate of up to 188 mmol h^−1^ g^−1^. The external quantum efficiencies were 30 % (5 min) and 14 % (75 min) at 450 nm. Furthermore, photo‐oxidation of methanol by Pdots, followed by a disproportionation reaction and an oxidation reaction, produced the high‐value chemical formate. On the basis of various spectroscopic and electrochemical measurements, the photophysical processes of the system were studied in detail and a reaction mechanism was proposed.

## Graphical abstract

H_2_O_2_ and formate were produced by employing organic polymer dots as the photocatalyst in alkaline conditions. The H_2_O_2_ production rate reached up to 188 mmol h^−1^ g^−1^ and EQEs were 30 % (5 min) and 14 % (75 min). A reaction mechanism is proposed.

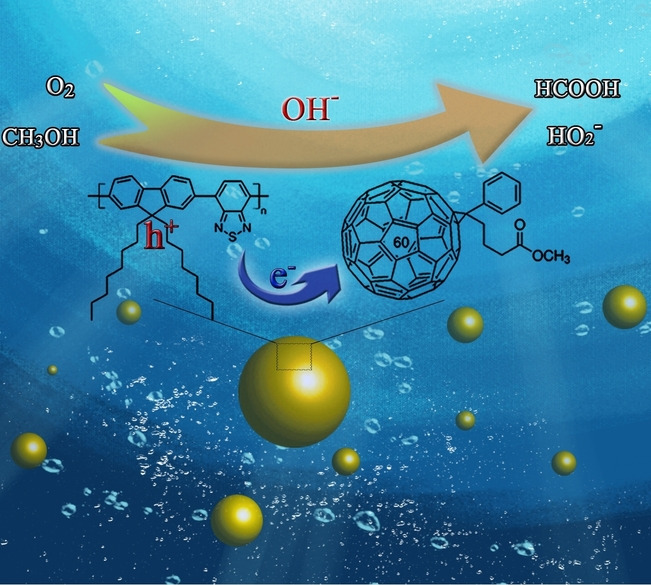

Hydrogen peroxide (H_2_O_2_) is a widely used industrial product for paper manufacturing, mining and water treatment.^[1]^ Moreover, the COVID‐19 is drawing up the demand of H_2_O_2_ as disinfection product due to the constantly spreading infectious disease and the demand is estimated to reach 5.7 million tons by 2027.^[1a]^ So far, 95 % of global industrial production of H_2_O_2_ is from anthraquinone (AQ) oxidation.^[2]^ The traditional AQ oxidation method unavoidably results in side reactions and products which are not environmentally benign, such as 2‐ethylanthraquinone, trioctyl phosphate and tert‐butyl urea.^[3]^ The constantly expanding demand of H_2_O_2_ will inevitably result in environment pollution and energy waste if the dependence on AQ method remains high. Photocatalytic H_2_O_2_ production using oxygen as the source, namely oxygen reduction reaction (ORR), and solar energy as the energy input is environmentally friendly and of promising potential in practical application.

Large efforts have been put in and numerous photocatalysis systems have been developed to efficiently produce H_2_O_2_ via ORR mechanism, such as In_2_S_3_@In_2_O_3_,^[4]^ C/Co_3_O_4_,^[5]^ Cu(acac)_2_/BiVO_4_ ^[6]^ and metal‐organic‐frameworks (MOFs).^[7]^ Unfortunately, although these materials show efficient photocatalytic performance in H_2_O_2_ production, they face adverse conditions where possible metal residuals after reaction are of risk of impurity and related high cost. In contrast, as a popular pure organic material, conjugated polymers bring merits such as tunable band gaps and non‐toxicity, which have also been used as photocatalysts for H_2_O_2_ production in various conditions such as in presence or absence of sacrificial reagents.^[8]^ Recently, making organic polymers into polymer dots (Pdots) has shown significant photocatalytic proton reduction performance as compared to bulk polymers.^[9]^ Heterojunction Pdots consisting of donor and acceptor components have been reported to have advantages in efficient charge separation which can further improve proton reduction reactions.^[10]^ However, to date, there is no work has been reported on H_2_O_2_ generation by use of Pdots. Moreover, few system has shown H_2_O_2_ generation in alkaline condition which actually is favorable in stabilizing H_2_O_2_ as well as for industrial applications.[^1a^, ^8c^]

In this work, we adopted heterojunction Pdots consisting of Poly(9,9‐dioctylfluorene‐alt‐benzothiadiazole) (PFBT) as an electron donor and 1‐[3‐(Methoxycarbonyl)propyl]‐1‐phenyl‐[6.6]C_61_ (PCBM) as an electron acceptor (Figure 1a) for light driven H_2_O_2_ production. PFBT has very good light absorption up to 550 nm and PCBM is an excellent electron acceptor which has shown satisfactory oxygen reduction reactivity.^[11]^ Energy levels of PFBT and PCBM (Figure 1a) allow feasible electron transfer from PFBT to PCBM upon light illumination, then performing O_2_ reduction reaction. The heterojunction binary PFBT‐PCBM system showed efficient H_2_O_2_ production in alkaline condition in presence of methanol and oxygen. The methanol (MeOH) can be converted into a higher‐valuable chemical formate by a photo‐oxidation reaction to formaldehyde followed by Cannizzaro reaction in alkaline condition and an oxidation reaction of formaldehyde in presence of H_2_O_2_. Various spectroscopic and electrochemical methods were employed to study the photophysical processes of the system to understand the reaction mechanism of H_2_O_2_ and formate formation.

**Figure 1:**
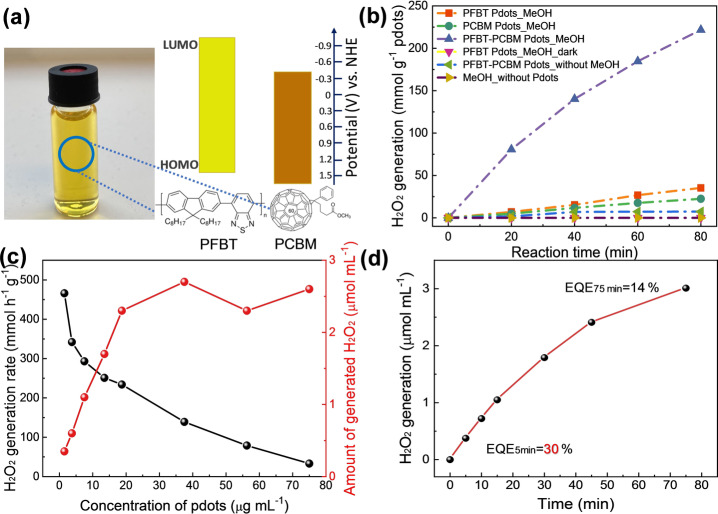
a) Molecular structures and energy levels of PFBT and PCBM and the photo of PFBT‐PCBM binary Pdots aqueous solution; b) Photocatalytic H_2_O_2_ generation experiment with PFBT Pdots, PCBM dots and PFBT‐PCBM binary Pdots under LED (420–750 nm, 50 mW cm^−2^) illumination with 3 mL of 20 μg mL^−1^ Pdots, 1 M KOH and 5 M MeOH; c) Photocatalytic H_2_O_2_ generation activity in 30 min with 3 mL PFBT‐PCBM binary Pdots in different concentrations under white LED illumination (50 mW cm^−2^, 420–750 nm); d) External quantum efficiency measurement with 3 mL 100 μg mL^−1^ PFBT‐PCBM Pdots, 1 M KOH and 5 M MeOH, excited at 450 nm, 2.9 mW cm^−2^.

PFBT‐PCBM binary Pdots were prepared by reported nanoprecipitation method and the experimental details can be found in Supporting Information. In order to evaluate photocatalytic H_2_O_2_ generation performance of PFBT‐PCBM Pdots, methanol was employed to take the photo‐generated holes from the Pdots to produce another useful chemical. In pH 14, PFBT‐PCBM Pdots (20 μg mL^−1^) showed H_2_O_2_ production of 188 mmol h^−1^ g^−1^ which was dramatically improved as compared to PFBT (26 mmol h^−1^ g^−1^) Pdots and PCBM (21 mmol h^−1^ g^−1^) dots (Figure 1b). The H_2_O_2_ generation process was proved to be photocatalytic reaction as no H_2_O_2_ was detected without any one of components (O_2_, Pdots, MeOH and light). Moreover, the similar reactivity (169 mmol h^−1^ g^−1^) of PFBT‐PCBM Pdots (24 μg mL^−1^) prepared from the washed‐PFBT (Pd residual less than 10 ppm) suggests that the organic components are indeed responsible for the catalytic reaction to form H_2_O_2_ (Figure S5).

The effect of Pdots concentration on photocatalytic performance was also investigated. As shown in Figure 1c, S6 and Table S2, the H_2_O_2_ generation rate on per unit mass of Pdots decreased from 466 to 33 mmol h^−1^ g^−1^ when the mass concentration of Pdots was increased from 1.5 to 75 μg mL^−1^. The plateau of total generated H_2_O_2_ (≈2.6 μmol mL^−1^ in 30 min) was achieved when Pdots concentration is over 20 μg mL^−1^, probably due to saturated light absorption or strong light scattering of Pdots at higher concentration. Therefore, the performance of H_2_O_2_ production on PFBT‐PCBM Pdots in concentration of 20 μg mL^−1^ was compared with and found to be superior to most of reported polymer‐based systems (Table S3). External quantum efficiencies (EQE, see Figure 1d) at 450 nm for the first 5 min and for 75 min reaction of PFBT‐PCBM Pdots were determined to be 30 % and 14 %, respectively, which are among the highest values reported so far.

The satisfactory photocatalytic performance indicates there must be efficient charge separation between PFBT and PCBM first to facilitate the following reactions. The steady‐state emission is therefore employed to perform fluorescence quenching experiments. The results show significant quenching of PFBT emission by PCBM in Pdots (Figure 2a). The reduction potential of PFBT (−1.0 V vs. Normal Hydrogen electrode (NHE)) is thermodynamically feasible to conduct electron transfer to PCBM with a reduction potential of −0.4 V vs. NHE.[^10b^, ^12^] Additionally, the absorption of PCBM at 500 to 680 nm is too weak to efficiently absorb photons emitted by excited PFBT (PFBT*) and perform energy transfer between PFBT and PCBM. Therefore, the quenching of PFBT emission by PCBM in Pdots should be dominantly caused by efficient electron transfer from PFBT* to PCBM.

**Figure 2:**
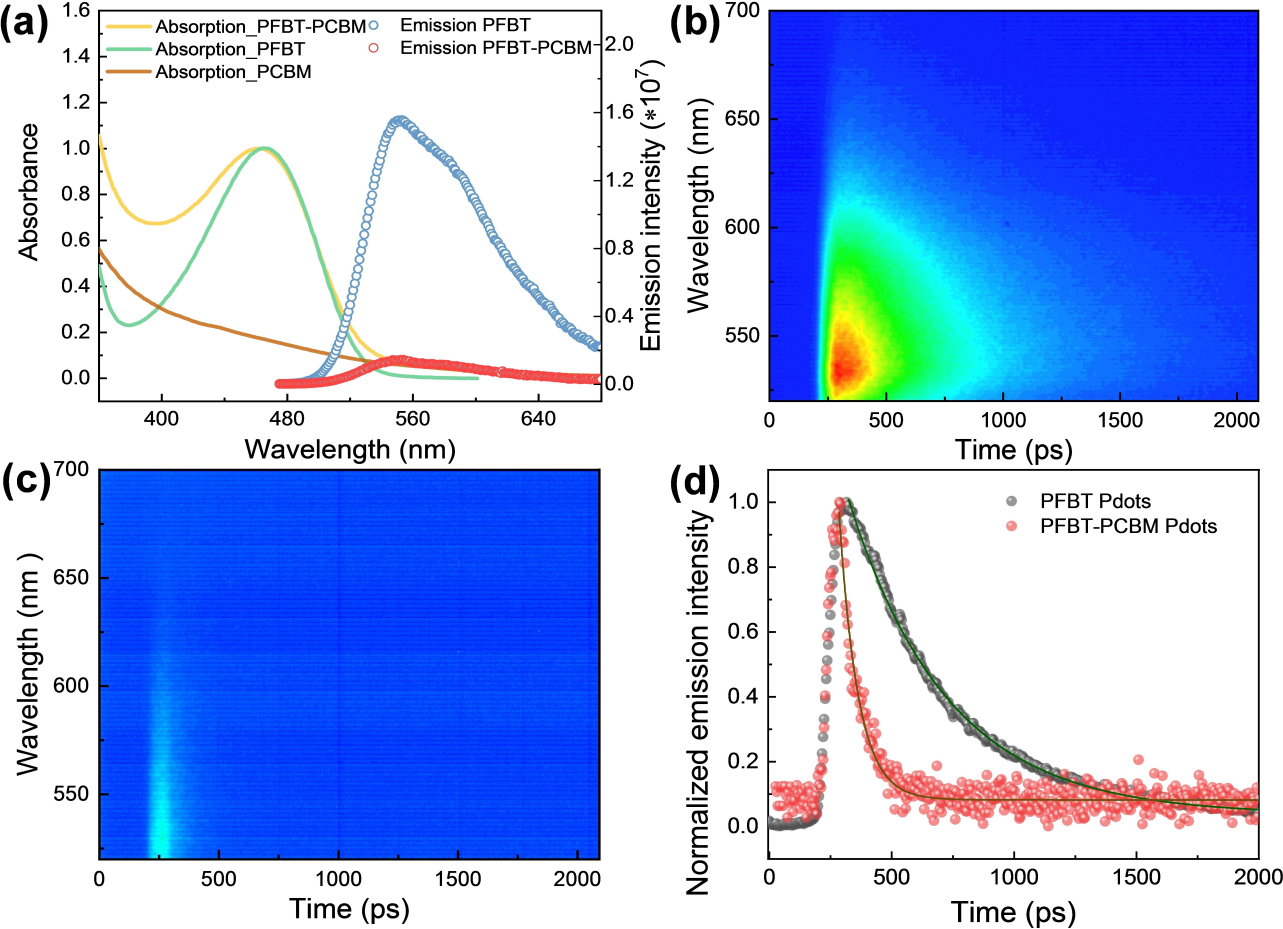
a) Steady‐state UV/Vis spectra of PFBT‐PCBM binary Pdots (yellow line), PFBT Pdots (green line) and PCBM dots (brown line) and fluorescence emission of PFBT‐PCBM binary Pdots (red circles) and PFBT Pdots (blue circles) in neutral aqueous solution. Fluorescence emission was excited at *λ*=450 nm; streak camera emission images of b) PFBT Pdots and c) PFBT‐PCBM Pdots in degassed neutral aqueous solution (color represents photon counts where red represents high and blue represents low), excited at 470 nm; d) fluorescence decay at 540 nm and relative mono‐exponential fits.

To further investigate the charge separation of this binary Pdots system, streak camera was used to further evaluate fluorescence lifetime of PFBT in singular (Figure 2b) and binary (Figure 2c) Pdots. The fluorescence lifetime (Figure 2d) of PFBT Pdots (*τ*_PFBT_) was determined to be 401±3 ps. When PCBM was added, the fluorescence lifetime of PFBT in the binary Pdots (*τ*_PFBT‐PCBM_) dramatically decreased to 80±2 ps, which is corresponding to a charge transfer efficiency (*η*_CT_) of 80 % (see calculation in Supporting Information).

In alkaline condition, the quenching of PFBT fluorescence in the binary Pdots is also found to be very efficient, up to 86 % (Figure S7). In contrast, there is no quenching effect observed in presence of MeOH. Therefore, the charge transfer between PFBT and PCBM should be the initial charge separation step in the PFBT‐PCBM binary Pdots when it is used for the photocatalytic reaction. The effect of Pd residual on electron transfer in PFBT‐PCBM Pdots was also excluded, which is discussed in Supporting Information (Figure S8, S9 and Table S4).

Notably, PFBT‐PCBM binary Pdots were found to be able to catalyze H_2_O_2_ generation only in alkaline condition (Figure S10). There are three possibilities: 1) methanol oxidation by oxidized PFBT (PFBT^+.^) was not feasible in low pH; 2) proton reduction competes with oxygen reduction reaction (ORR); 3) generation of singlet oxygen (^1^O_2_) is more dominant in low pH.^[13]^

According to streak camera results discussed above, the charge transfer occurred between PFBT and PCBM upon light illumination is efficient and ultrafast, which results in formation of PFBT^+.^ and reduced PCBM (PCBM^−.^). Methanol oxidation was reported to have significantly higher reactivity in alkaline compared to neutral and acidic conditions.^[14]^ To verify this in our system, photoelectrochemical measurements were designed and carried out in various pH conditions (Figure 3 and S11). The object used in this study was a mesoporous TiO_2_ film on FTO coated with PFBT. Photo‐generated electrons of PFBT can be thermodynamically transferred to the conduction band (CB) of TiO_2_ (−0.5 V vs. NHE) and then form PFBT^+.^. As shown in Figure S11, in pH 14, an oxidation current starts at ca. 0.66 V vs. NHE was assigned to MeOH oxidation. The contribution of photo‐generated PFBT^+.^ was intuitively proved by operating a photoelectrochemical measurement with chopped light (Figure 3). The current density of MeOH oxidation increased dramatically upon illumination. In neutral and acidic conditions, no MeOH oxidation peak or photoactivity was observed, indicating that MeOH oxidation is only occurred in alkaline condition by PFBT polymer.

**Figure 3:**
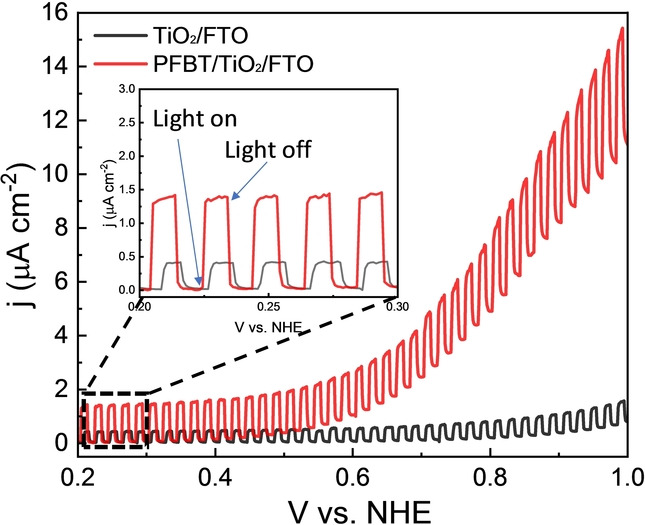
Photoelectrochemical measurements in 1 M KOH, 5 M MeOH solution with PFBT polymer dropcast on mesoporous TiO_2_ film, scan rate of 1 mV s^−1^, light chopped every 10 s.

In order to check if the proton reduction competes with ORR in alkaline condition, photocatalytic H_2_ generation was conducted at different pH with the PFBT‐PCBT Pdots. As shown in Figure S12, no H_2_ was detected during 1500 s, suggesting proton reduction can be ruled out as a competing process to ORR to inhibit H_2_O_2_ production in low pH.

If singlet oxygen (^1^O_2_) was formed during photocatalysis via photoreduction of oxygen (O_2_) into oxygen superoxide (O_2_^.−^) by PFBT* and/or PCBM^−.^ followed by an oxidation of O_2_^.−^ by PFBT^+.^, then photogenerated electrons and holes in PFBT will not be completely used for H_2_O_2_ formation. To check if ^1^O_2_ formation is really a competing process to H_2_O_2_ formation, 9,10‐Anthracenediyl‐bis(methylene)dimalonic acid (ABDA) was used as the probe of ^1^O_2_ because it is degraded by selectively reacting with ^1^O_2_.[^12^, ^15^] It is facile to monitor the absorption of ABDA and the degradation rate of ABDA can reflect the generation rate of ^1^O_2_ in the reaction. As shown in Figure 4a, ^1^O_2_ generation rate obviously increased along with the decrease of pH (Figure 4a), implying the efficient ^1^O_2_ generation in low pH consumes holes in PFBT^+.^ probably due to inefficient oxidation of MeOH. By conducting experiments in alkaline, a strong evidence shows that ^1^O_2_ generation can be dramatically inhibited when system contains MeOH (Figure 4b). However, the ^1^O_2_ generation is still competing with oxidation of MeOH in alkaline condition. With photocatalytic H_2_O_2_ generation reaction proceeded, reactivity of PFBT‐PCBM binary Pdots continuously decreased together with its absorbance (Figure S13) and hydrodynamic size (Figure S14). O_2_^.−^ ^[8a]^ and ^1^O_2_ ^[16]^ have been reported to degrade polymers. In our system, according to XPS data (Figure S15), the degradation of PFBT‐PCBM system is probably through the opening of thiadiazole rings in PFBT polymers caused by the photogenerated ^1^O_2_ as discussed in Supporting Information. The generated H_2_O_2_ does not degrade Pdots as evident by a control experiment (Figure S16). Therefore, kinetically increasing MeOH oxidation by Pdots to inhibit ^1^O_2_ formation could further stabilize the Pdots system and further improve H_2_O_2_ production.

**Figure 4:**
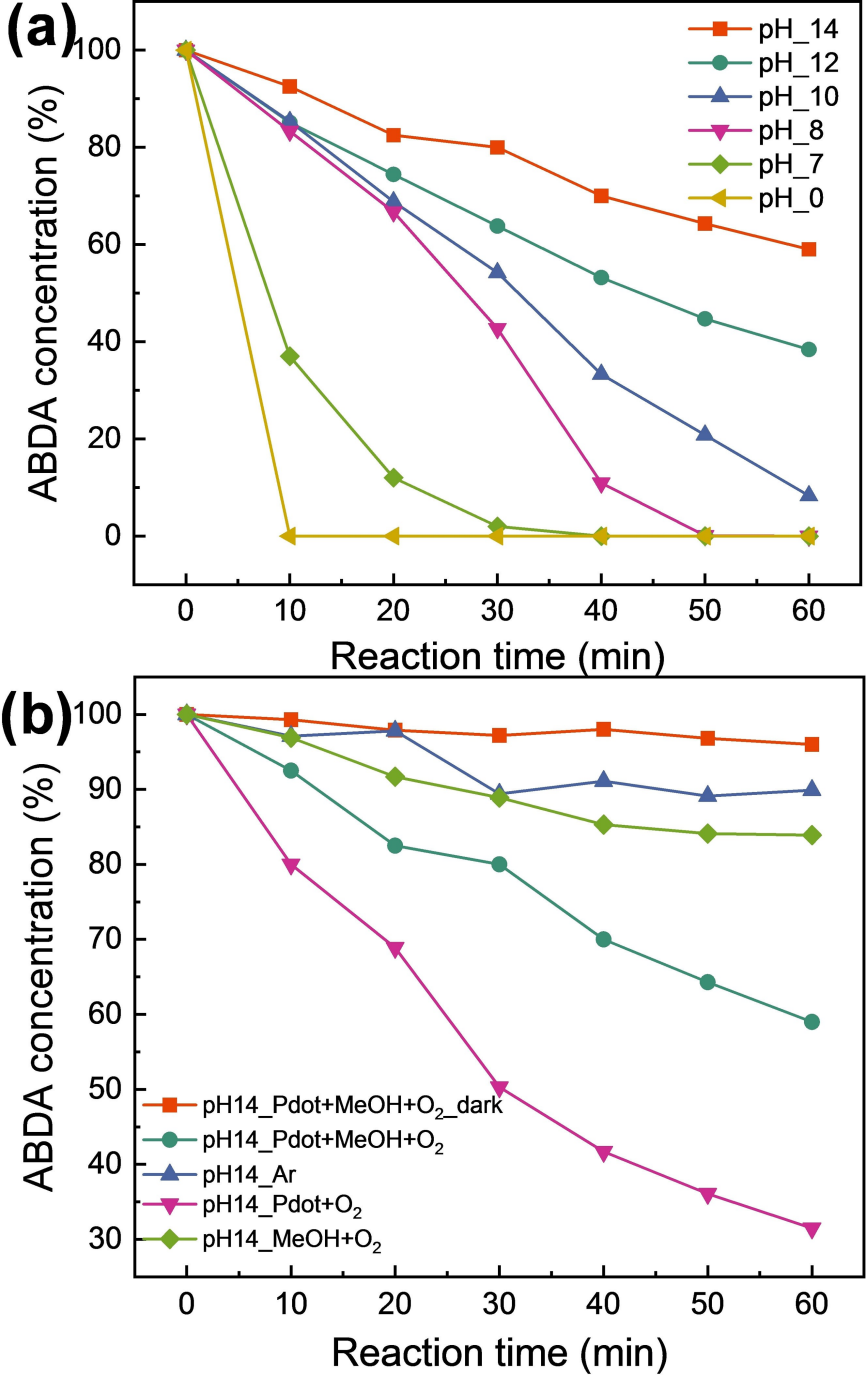
a) ABDA degradation with PFBT‐PCBM binary Pdots of 20 μg mL^−1^ in 5 M MeOH, various pH controlled by adjusting concentration of KOH or HCl. b) ABDA degradation with PFBT‐PCBM binary dots of 20 μg mL^−1^ in 1 M KOH with various conditions such as reaction in the dark, without MeOH and purging with Ar.

In order to figure out the product of methanol oxidation in our system, nuclear magnetic resonance (NMR) spectroscopy was employed. As shown in Figure 5, a signal appeared at chemical shift of 8.3 ppm in ^1^H NMR of solution after reaction was assigned to the proton in formate in alkaline condition.^[17]^ The existence of reaction between HCOO^−^ and H_2_O_2_ was excluded (Figure S17) and consequently the mole ratio of generated formate and H_2_O_2_ can be calculated to explore the possible reaction mechanism. The mole of the produced formate was estimated to be 9 μmol, and the generated H_2_O_2_ was 8 μmol. As the MeOH should be photo‐oxidized to formaldehyde first (as discussed below), the result suggests that the formation of formate could be contributed by formaldehyde oxidation with H_2_O_2_ and a disproportionation reaction between two formaldehyde molecules (known as Cannizzaro reaction).

**Figure 5:**
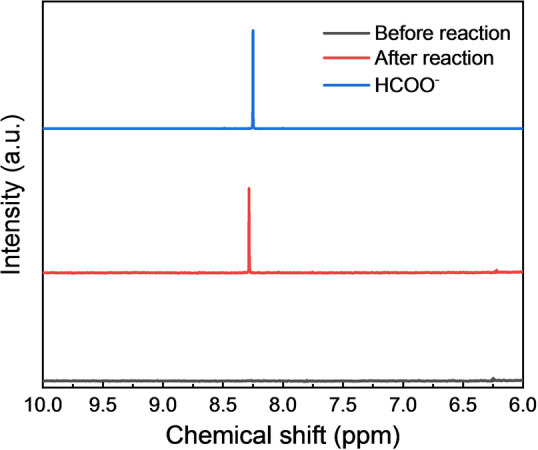
^1^H NMR spectra of formate aqueous solution and the solution before and after photocatalytic H_2_O_2_ generation with PFBT‐PCBM Pdots.

On basis of all experimental results, methanol oxidation, oxygen reduction and alkaline condition are the three necessary criteria for photocatalytic H_2_O_2_ and formate generation by the PFBT‐PCBM binary Pdots. Therefore, a photocatalytic reaction process is proposed (Scheme 1). Briefly, after charge separation, PCBM^−.^ and PFBT^+.^ are formed. One O_2_ molecule is reduced by PCBM^−.^ to form O_2_^.−^ and the deprotonated methanol molecule is oxidized by PFBT^+.^ to produce CH_3_O⋅ in alkaline condition. The CH_3_O⋅ reacts with O_2_^.−^ to form an unstable intermediate which has rearrangement to produce deprotonated hydrogen peroxide (HO_2_^−^) and formaldehyde (CH_2_O). The participation of Pdots during this process should not be excluded. Subsequently, two possible pathways generate formate and result in H_2_O_2_ and formate with 1 : 1 ratio observed under our photocatalytic condition: i. two CH_2_O molecules go through Cannizzaro reaction (Figure S18) in alkaline condition and produce one molecule of MeOH and formate respectively; ii. CH_2_O is oxidized by H_2_O_2_ to produce formate (Figure S19).

**Scheme 1:**
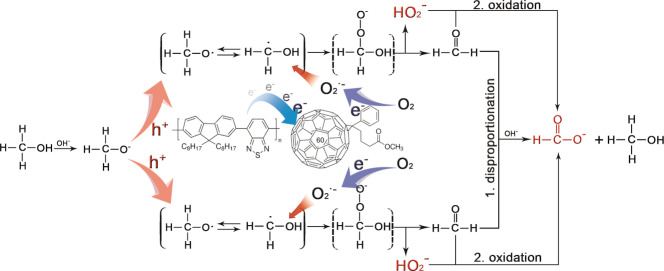
The proposed photocatalytic H_2_O_2_ and formate production with PFBT‐PCBM binary Pdots.

In summary, a binary Pdots photocatalyst consisting of organic polymer PFBT as the electron donor and PCBM as the electron acceptor was employed for light‐driven H_2_O_2_ and formate production in alkaline condition. The PFBT‐PCBM binary Pdots exhibited a reactivity for H_2_O_2_ production up to 188 mmol h^−1^ g^−1^ with a concentration of Pdots 20 μg mL^−1^ and EQE of 30 % (5 min) and 14 % (75 min) at 450 nm. A reaction mechanism for the photocatalytic H_2_O_2_ and formate formation by the PFBT‐PCBM binary Pdots is proposed and shows that the formation of H_2_O_2_ and formate goes through photoreduction and photooxidation reactions followed by combination of a disproportionation reaction and an oxidation reaction. This work may inspire further design of photocatalytic systems for simultaneous photocatalytic generation of the clean fuel H_2_O_2_ and other value‐added chemicals.

## Conflict of interest

The authors declare no conflict of interest.

## Supporting information

As a service to our authors and readers, this journal provides supporting information supplied by the authors. Such materials are peer reviewed and may be re‐organized for online delivery, but are not copy‐edited or typeset. Technical support issues arising from supporting information (other than missing files) should be addressed to the authors.

Supporting Information

## Data Availability

The data that support the findings of this study are available from the corresponding author upon reasonable request.
