# Supplementary material for: Efficient Generation of Hydrogen Peroxide and Formate by an Organic Polymer Dots Photocatalyst in Alkaline Conditions
Source: Angew Chem Int Ed Engl. 2022 Apr 5;61(23):e202202733. doi: 10.1002/anie.202202733 (PMC9324198; doi:10.1002/anie.202202733)
Supplement: Supplementary file 1 — Supporting Information [file ANIE-61-0-s001.pdf]

Angewandte Chemie
Eine Zeitschrift der Gesellschaft Deutscher Chemiker

Supporting Information

Efficient Generation of Hydrogen Peroxide and Formate by an Organic Polymer Dots Photocatalyst in Alkaline Conditions

*S. Wang, B. Cai, H. Tian**

Supporting information

Materials

Poly[(9,9-dioctylfluorenyl-2,7-diyl)-co-(1,4-benzo-{2,1',3}- thiadiazole)] (PFBT, MW 250000) was purchased from Ossila, UK. The co-polymer, polystyrene grafted with ethylene oxide and carboxyl groups (PS-PEG-COOH, back bone chain MW 8500, graft chain MW 4600, total chain MW 36500), was purchased from Polymer Source Inc., Canada. All other reagents were purchased from Sigma-Aldrich (Sweden) and used as received unless indicated otherwise. All experiments and measurements were carried out at room temperature unless indicated otherwise. The washed PFBT with low Pd (<10 ppm) is provided from Brilliant Matters, Canada.

Preparation of PFBT, PCBM and PFBT-PCBM Pdots

Pdots were prepared via our previously reported method.[1] Taking PFBT Pdots as an example, PFBT and PS-PEG-COOH were separately dissolved in THF to make stock solution in concentration of 0.05 mg mL^{-1} and 1 mg mL^{-1} respectively. Then, 5 mL PFTB solution and 1 mL PS-PEG-COOH were mixed and treated by sonication for 20 min. The mixture was poured into 20 mL deionized water and then the formed dark yellow solution was sonicated for 20 min in order to ensure the homogeneity. At last, the homogenous solution was

left in the dark over night to completely evaporate THF and pure PFBT Pdots were obtained afterwards.

Photocatalytic H_2O_2 generation

Photocatalytic H_2O_2 generation reaction was proceeded in a quartz cuvette. In detail, 3 mL Pdots reaction solution (20 μg mL^{-1}) which contained 5 M MeOH and 1 M KOH was purged by O_2 for 30 min in order to ensure the saturation of O_2 in solution. Afterwards, the cuvette was irradiated by a LED lamp (420 - 750 nm) with irradiation density of 50 mW cm^{-2}.

Detection of H_2O_2

Photocatalytic generated H_2O_2 was detected by a widely used DPD (N, N-diethyl-p-phenylenediamine) method.[2] Typically, 200 μL reaction solution was collected from reaction cuvette and mix with 2 mL phosphate buffer solution (pH 5.4). Then, 50 μL of DPD (10 mg mL^{-1}) and 50 μL of POD (1 mg mL^{-1}) solution were added into the mixture successively. The mixture was stored in the dark for 1 min for complete reaction. The resulted red solution was used to detect concentration of H_2O_2 by the absorption at 551 nm, calibration plots are shown in Figure S1.

Detection of $^1O_2$

9,10-Anthracenediyl-bis(methylene)dimalonic acid (ABDA) was used as the probe for $^1O_2$. For measurement in alkaline condition, aqueous solution which contained 0.1 mg/mL ABDA, 20 μg mL^{-1} Pdots, 5 M MeOH and 1 M KOH was purged with O_2 and then was placed under illumination of a LED lamp (420 nm $\leq\lambda\leq$750 nm) with irradiation density of 50 mW cm^{-2}. The generation rate of $^1O_2$ was indirectly measured by the decrease of the absorbance at 405 nm.

Detection of H_2

Hydrogen in solution was detected by using a Unisense microsensor in the same condition of photocatalytic H_2O_2 production test.

Dynamic Light Scattering (DLS) Measurements

The hydrodynamic diameter of samples was measured by a Zetasizer Nano-S from Malvern Instruments Nordic AB.

Steady-State Absorption and Fluorescence Measurements.

Steady-state UV−vis measurements were analyzed by using a PerkinElmer Lambda 750 UV−vis spectrophotometer. Steady-state fluorescence spectra were analyzed by using a Fluorolog 3-222 emission spectrophotometer (Horiba Jobin-Yvon) together with the FluorEssence software.

Time-resolved fluorescence lifetime measurement

The fluorescence decays of all samples were recorded using a streak camera/spectrograph combination (Hamamatsu, $\leq$ 5 ps fwhm) and 470 nm, 100 fs pulse was used for excitation.

Photo-electrochemical measurements

TiO_2 firstly grown onto FTO glasses followed by the calcination at 573 K for 1h and then cooled down to room temperature. Afterwards, a film of PFBT was spin-coated onto TiO_2 film with PFBT solution in CH_2Cl_2. The as-prepared FTO was then employed as the working electrode for PEC methanol oxidation test, Ag/AgCl and Pt wire worked as reference and counter electrode respectively. For test in solutions of pH 14, 7 and 0, 1 M KOH, 1 M KCl and 1 M HCl solution were respectively employed as the electrolytes, linear sweep voltammetry (LSV) method was used to scan from 0 to 1 V vs. NHE at scan rate of 50 mV s^{-1}. In Chopped LSV measurement, the scan rate was 1 mV s^{-1}.

NMR measurements

Proton nuclear magnetic resonance spectra (1 H NMR) were recorded on a Varian FT-NMR spectrometer (400 MHz for 1 H NMR). Pdots prepared in D_2O were used. Typically, the reaction conditions were the same as photocatalytic activity test except that KOH aqueous solution and MeOH were replaced by KOH D_2O solution and CH_3OD respectively. After reaction, 0.6 mL reaction solution was extracted from the cuvette for NMR measurement.

For the experiments of Cannizzaro reaction, the same system as above was used except the existence of 2 mg mL^{-1} HCHO (typically in the form of low polymer where the n is from 2 to 8).[3] Samples were maintained for 24 h for completed reaction because the depolymerisation of HCHO is slow without heating.

Quantum Yield Measurement

External quantum efficiency (EQE) of PFBT-PCBM dots was measured under the same condition as for photocatalytic H_2O_2 generation except that the concentration of Pdots was 100 µg mL^{-1}. The light source was replaced by Xenon lamp with light filter (QD 450 nm) and the irradiation density was 2.9 Mw cm^{-2}. EQE was calculated by the equation below:

$$\mathrm{EQE} = \frac{2 * generated\ H_2O_2\ molecules}{incident\ photons\ (N)}$$

$$\mathrm{N} = \frac{It\lambda}{hc}$$

Where N represents the incident photons, *I* represents the irradiation density, t is the irradiation time, λ is the wavelength of incident light, h is the plank constant and c is the speed of light.

X-ray photoelectron spectroscopy (XPS) measurement

X-ray photoelectron spectra (XPS) were recorded by using the instrument PHI Quantera II from Physical Electronics. For sample preparation, both samples before and after reaction was dried by being exposed in fume hood for 48 h. The MeOH and KOH were removed by dialysis for 48 h with 1.5 L dialysis water changed for 3 time. The dried samples were then dissolved by CH_2Cl_2 and drop casted onto ITO glass before XPS measurement.

Inductively coupled plasma mass spectrometry (ICP-MS) measurement

ICP-MS was measured by Brilliant Matters Inc., Canada with the details given below:

ICP-MS/MS (Model 8900, Agilent Technologies, Mississauga, ON, Canada) was used to measure (list of Elements) and any potential stable interferences. Liquid samples were injected into the plasma at a flowrate of 400 μL min^{-1} using a sample introduction system composed of a high efficiency concentric Micromist nebulizer (SCP Science, Canada) attached to a double-pass spray chamber maintained at 2℃. The optimized parameters are given in Table S1.

Table S1: ICP-MS/MS parameters

Parameters	Conditions
Sampler Cone	Nickel
Skimmer Cone	Nickel
Extraction Lens	S-Lens
RF Power	1500 W
RF Matching	1.80 V
Sampling Depth	7.0 mm
Carrier Gas flow rate	0.80 L/ Min
Makeup Gas flow rate	0.34 L/ Min
Spray Chamber temperature	2°C
Extract 1	8 V
Extract 2	1 V
Omega Bias	-200 V
Omega Lens	27.5 V
Q1 Entrance	0 V
Q1 Exit	1 V
Cell Focus	-2 V
Cell Entrance	-48 V
Cell Exit	-60 V
Deflect	-14.8 V
Plate Bias	-40 V
Q1 Bias	1 V
Q2 Bias	-15 V
Gas	No gas
Expected Oxide level	0.5 - 1 %
Expected Double Charged ions	0.3 - 0.5 %
Integration time/Mass	11.52 sec
m/z monitored (Q_1 and Q_2)	210

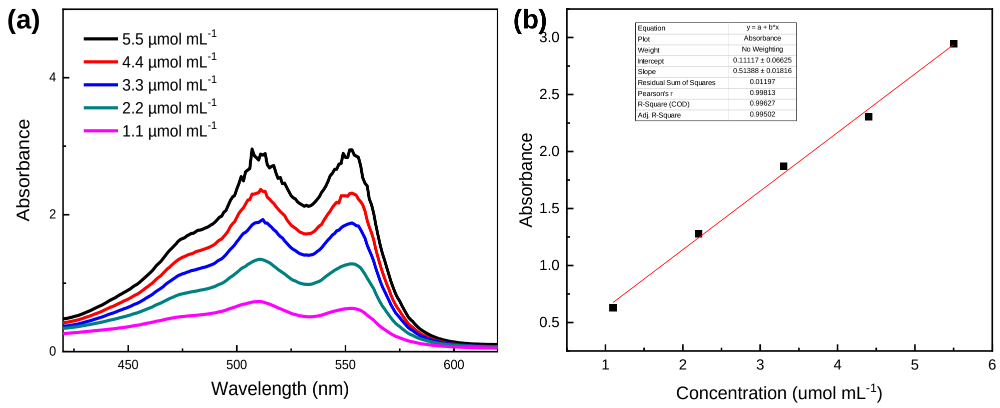

Figure S1. Calibration plots of H_2O_2 concentration in 5 M MeOH and 1 M KOH solution.

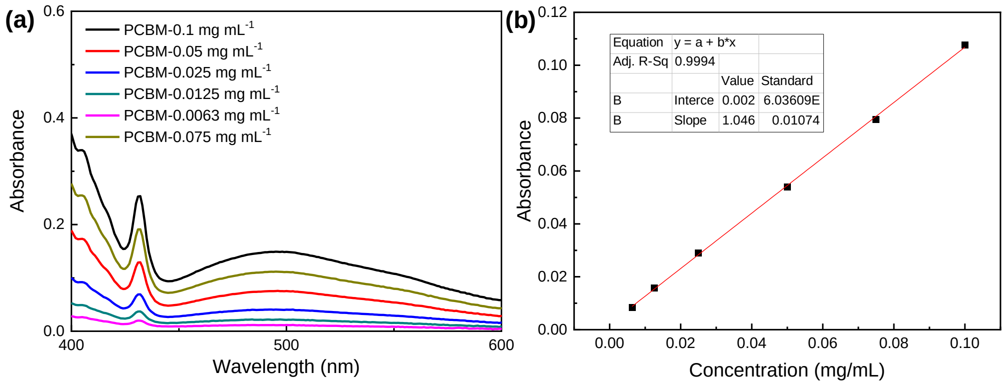

Figure S2. Calibration plots of different concentration of PCBM Pdots.

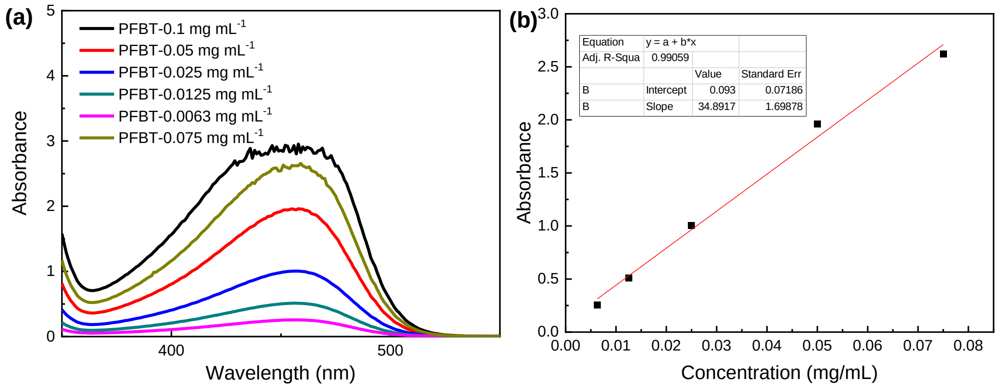

Figure S3. Calibration plots of different concentration of PFBT Pdots.

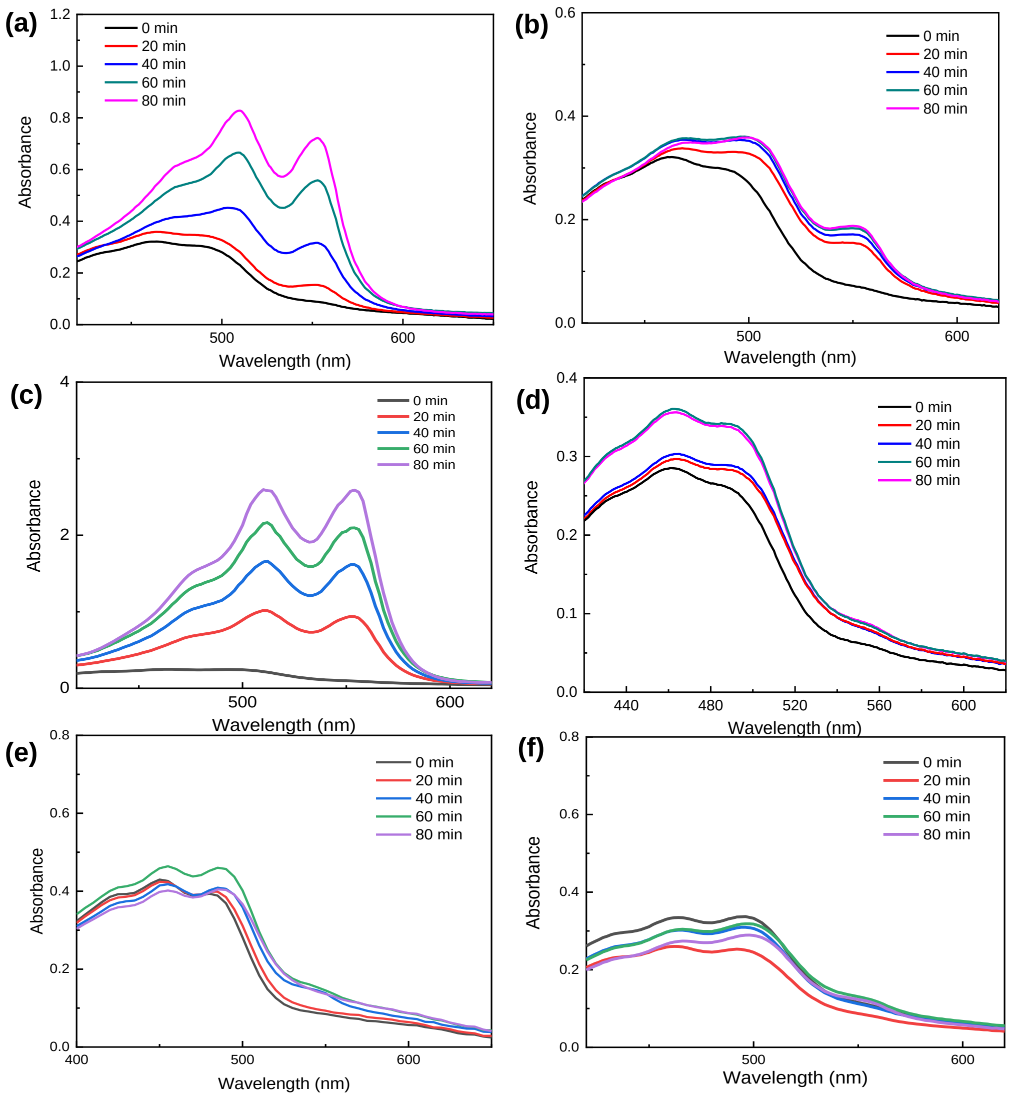

Figure S4. H_2O_2 generation test with (a) PFBT Pdots, (b) PCBM Pdots, (c) PFBT-PCBM Pdots, (d) PFBT Pdots in the dark, (e) PFBT-PCBM Pdots without MeOH and (f) system without Pdots.

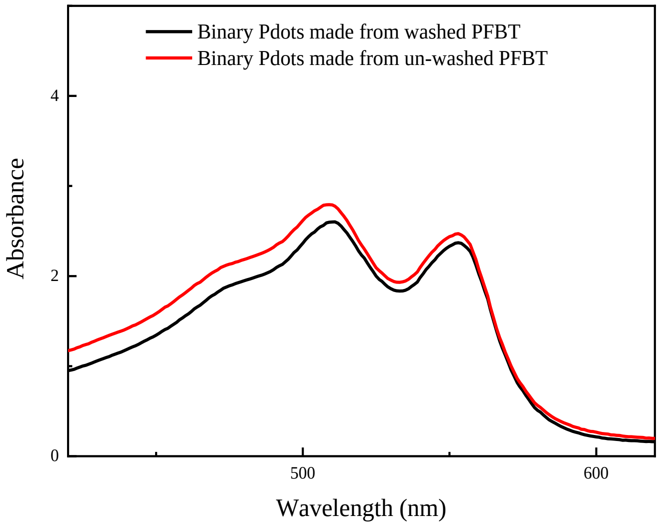

Figure S5. H_2O_2 generation test in 60 min with PFBT-PCBM Pdots prepared from washed and un-washed PFBT.

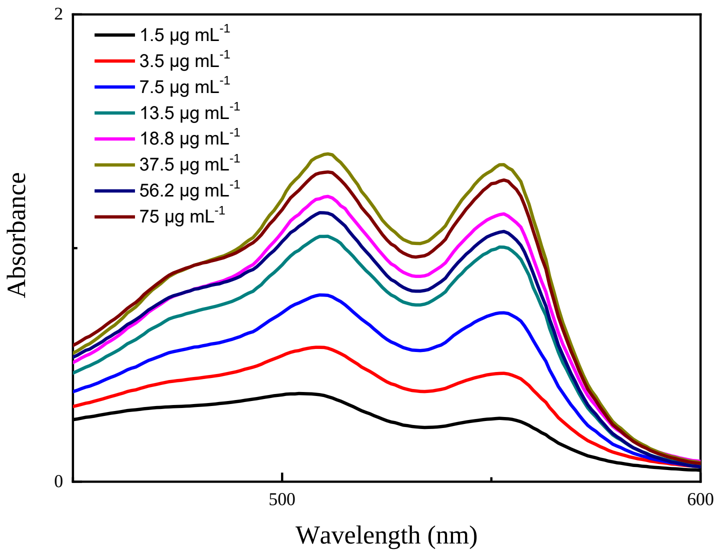

Figure S6. H_2O_2 generation with different mass concentration of PFBT-PCBM Pdots.

Table S2. Summary of H_2O_2 generation with PFBT-PCBM binary Pdots in various concentrations in 30 min.

Concentration of Pdots ($\mu g\ mL^{-1}$)	Catalytic performance ($\mu mol\ H_2O_2 \cdot mL^{-1}$)	Catalytic performance ($mmol\ H_2O_2 \cdot h^{-1} \cdot g^{-1}$)
1.5	0.35	466
3.5	0.6	342
7.5	1.1	293
13.5	1.7	251
18.8	2.2	234
37.5	2.7	139
56.2	2.3	79
75	2.6	33

Table S3. Recent works of polymer-based photocatalysts for H_2O_2 production.

Sample	Light source	Activity (mmol h^{-1} g^{-1})	EQE	Ref.
KPF_6-g-C_3N_4	Xenon lamp (300W) λ ≥420 nm	0.67	24.3 % at 420 nm	[4]
N_{vac}-g-C_3N_4	Xenon lamp (300W) λ ≥420 nm	1.84	3.2 % at 420 nm	[5]
Nf-SNG/TiO_2	Xenon lamp (500W) λ ≥420 nm	14.9	-	[6]
CeO_2/g-C_3N_4	Xenon lamp (300W) λ ≥420 nm	3.04	-	[7]
g-C_3N_4 nanosheets	Simulated sunlight	0.66	4.3 % at 420 nm	[8]
P-g-C_3N_4 nanosheets	Simulated sunlight	1.1	0.0018 % at 630 nm	[9]
O-g-C_3N_4	Xenon lamp (300W) λ ≥420 nm	3.02	10.2 % at 420 nm	[10]
g-C_3N_4 nanosheet	Xenon lamp (300W) λ ≥420 nm	1. 08	0.88 % at 365 nm	[11]
IO g-C_3N_4	Xenon lamp (300W) λ ≥420 nm	0.651	-	[12]
PFBT-PCBM dots	LED 420 nm ≤λ≤750 nm	188	14% (75 min) and 30% (5 min) at 450 nm	This work

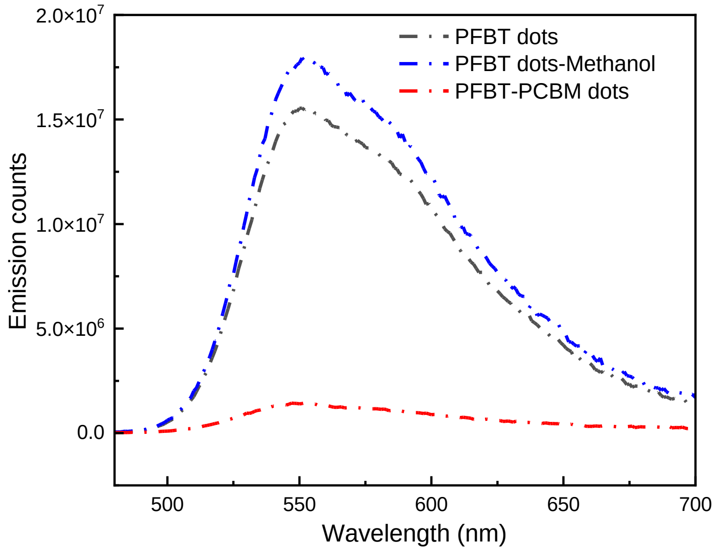

Figure S7. Fluorescent emission of PFBT Pdots, PFBT-PCBM Pdots and PFBT Pdots with MeOH in pH 14.

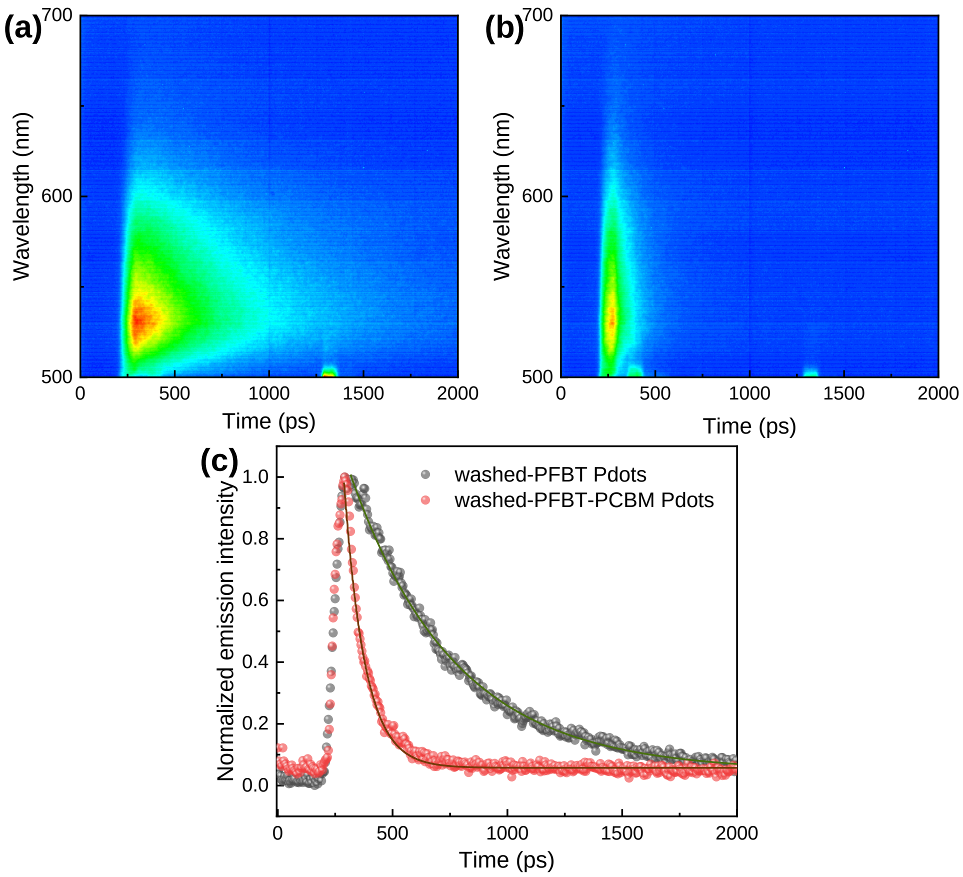

Figure S8. Streak camera emission images of (a) washed-PFBT Pdots and (b) washed-PFBT-PCBM Pdots (color represents photon counts where red represents high and blue represents low); (c) fluorescence decay at 540 nm and relative mono-exponential fits.

Streak camera was conducted to deeply investigate the potential effect of Pd to charge transfer. As shown in Figure S7, fluorescence lifetime of washed-PFBT dots and washed-PFBT-PCBM Pdots were measured to be 453 ± 4 and 91 ± 1 ps, respectively, which was similar to PFBT dots and PFBT-PCBM dots. According to equ. (1) and (2), the charge transfer efficiency (η_{CT}) and charge transfer rate (τ_{CT}^{-1}) was calculated to be 80 % and 8.5 × 10^9, respectively. Considering the hydrodynamic sizes of these two binary dots were also similar (Figure S9), we can have the conclusion that Pd residuals did not have stark

implication on charge transfer or photocatalytic performance of PFBT-PCBM Pdots in this study.

$$\eta_{CT}=1-\frac{\tau_{PFBT\text{-}PCBM}}{\tau_{PFBT}} \quad (1)$$

$$\frac{1}{\tau_{PFBT\text{-}PCBM}}=\frac{1}{\tau_{CT}}+\frac{1}{\tau_{PFBT}} \quad (2)$$

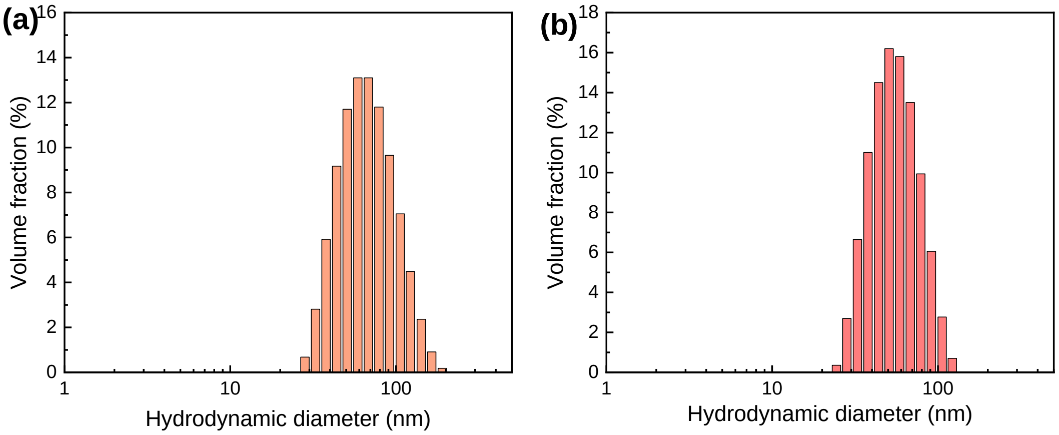

Figure S9. DLS measurement of (a) PFBT-PCBM Pdots and (b) washed-PFBT-PCBM Pdots.

Table S4. Comparison of properties of PFBT-PCBM binary dots made from washed- or unwashed-PFBT.

Sample name	H_2O_2 generation rate (mmol h^{-1} g^{-1})	η_{CT} (%)	τ_{CT} ($\times10^{10}$)	Size (nm)
PFBT-PCBM dots	188	80	1	70
Washed-PFBT-PCBM dots	169	80	0.85	60

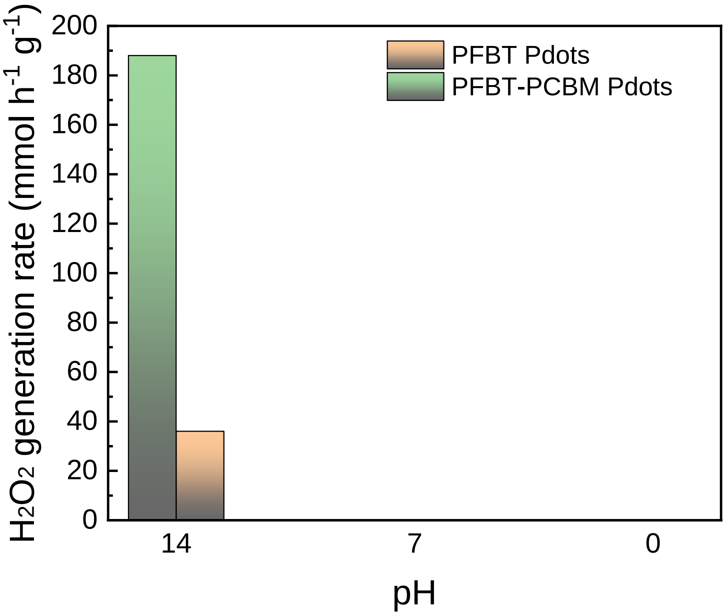

Figure S10. Photocatalytic H_2O_2 generation with PFBT and PFBT-PCBM Pdots in pH14, 7 and 0.

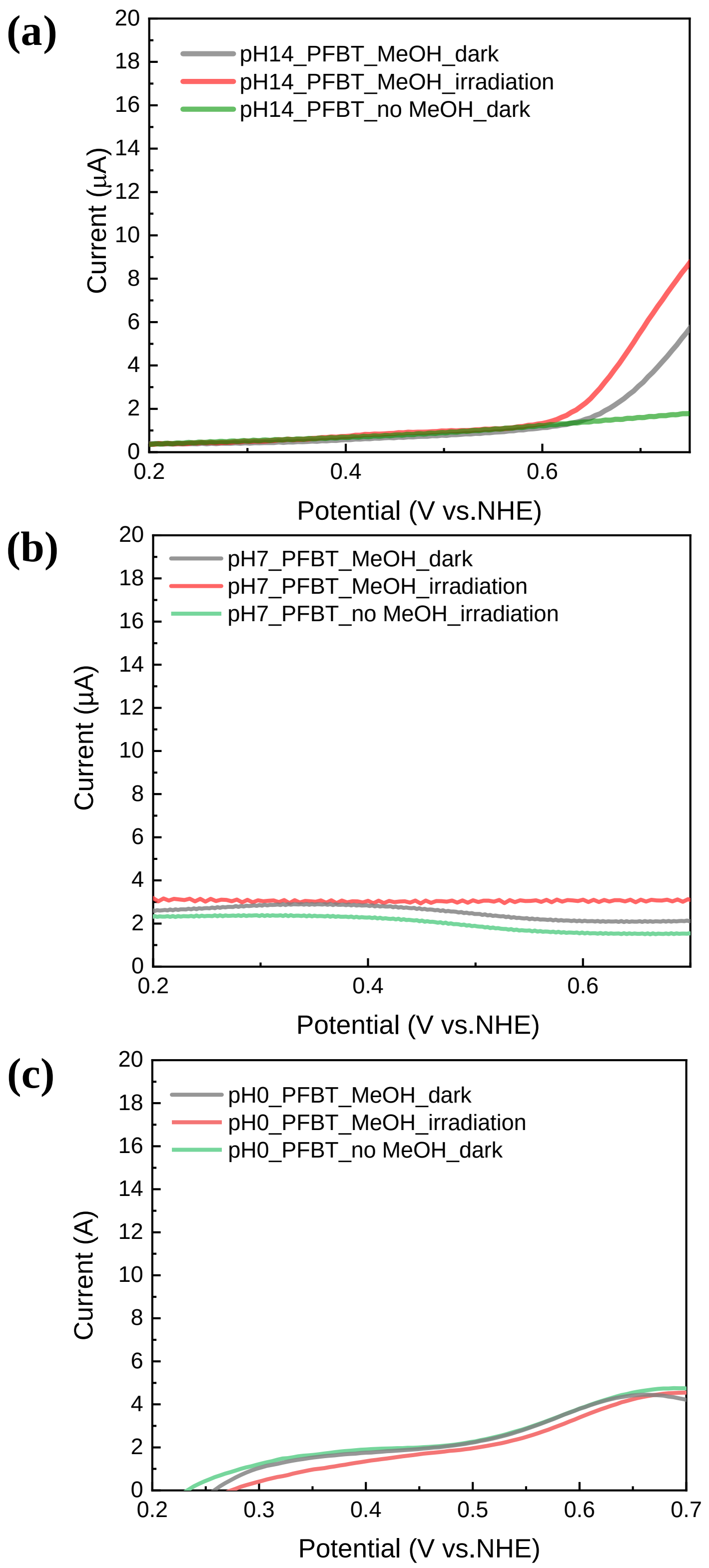

Figure S11. Methanol oxidation on FTO glass coated with TiO_2 and PFBT in (a) alkaline, (b) neutral and (c) acidic conditions.

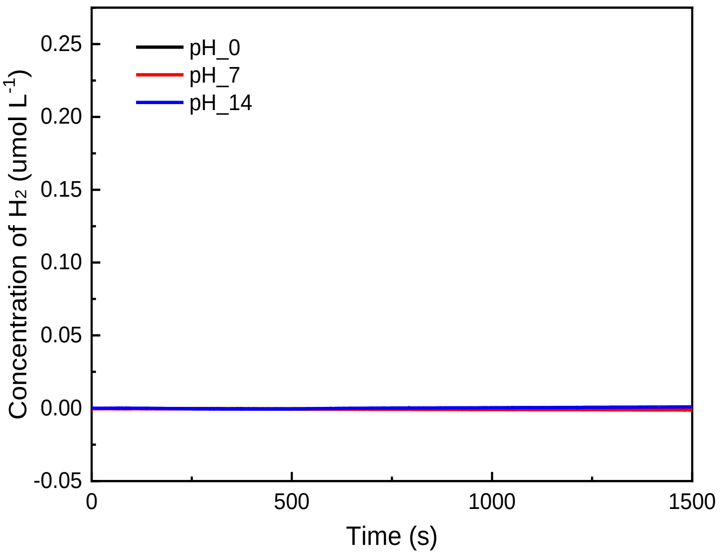

Figure S12. Photocatalytic H_2 evolution test with PFBT-PCBM Pdots under alkaline, neutral and acidic conditions.

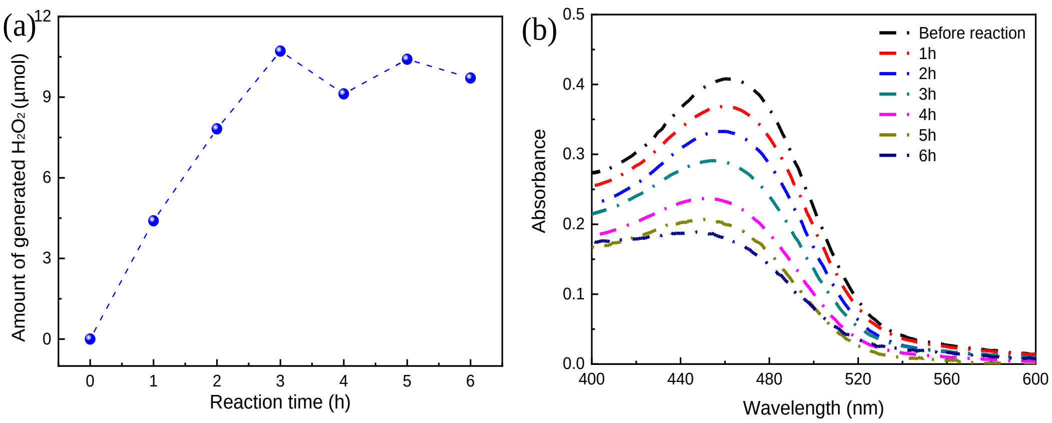

Figure S13. (A) H_2O_2 generation with PFBT-PCBM Pdots in 6 hrs; (B) Change of absorbance of pdots in reaction process.

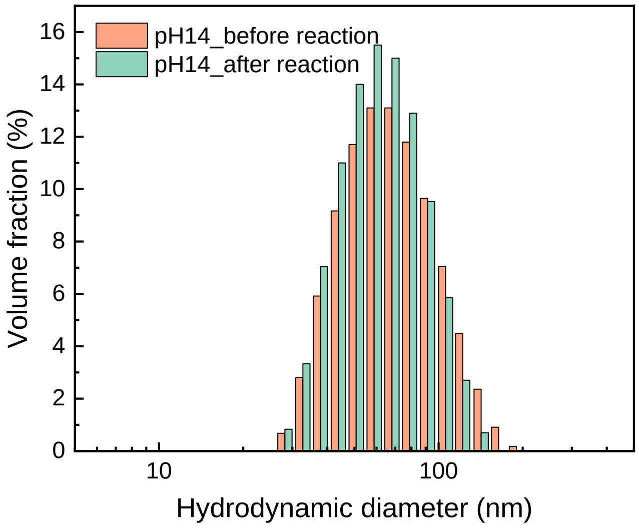

Figure S14. DLS measurement of PBFT-PCBM dots before and after 4h reaction.

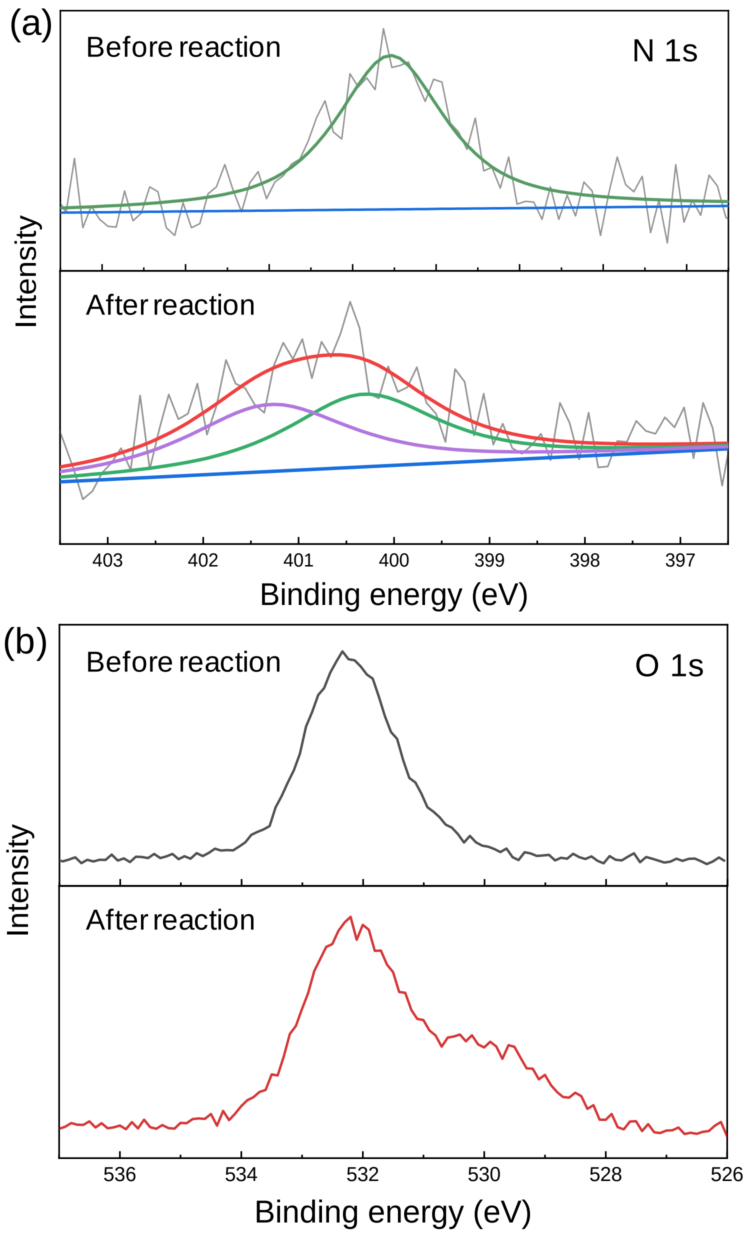

Figure S15. High resolution N 1s (a) and O 1s (b) XPS spectra of PFBT-PCBM Pdots before and after reaction.

The perfectly fitted single peak at 400.0 eV in N 1s spectrum is attributed to C=N-S since it is the only N component in PFBT-PCBM Pdots. After reaction, the shape of the peak become obviously broader and the new peak is fitted at 401.2 eV which can be resulted from the charge redistribution after the cleavage of thiadiazole rings. The hetero-atoms were probably oxidized by $^{1}O_2$ after or before the ring cleavage. This conclusion is further supported by O 1s

spectra where a new peak generated at 530.0 eV after reaction which can originate from the generation of O-N and O-S bonds. Therefore, the degradation of PFBT-PCBM Pdots during reaction process mainly occured on cleavage of thiadiazole rings and consequently the oxidation of N and S atoms.

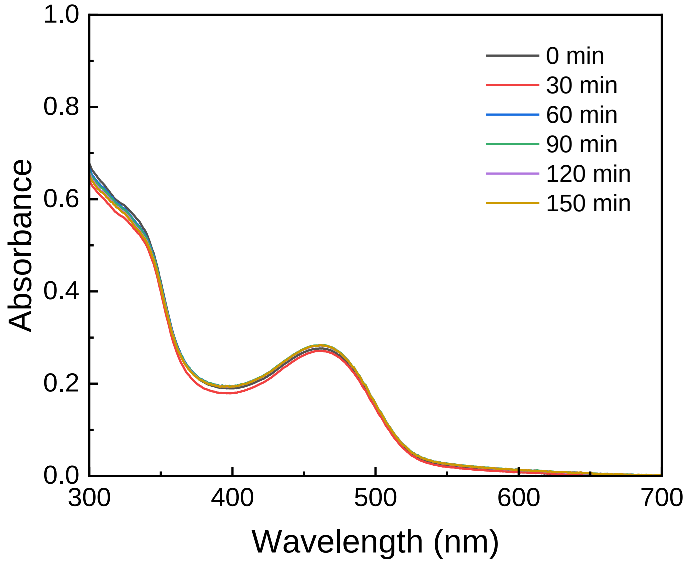

Figure S16. Stability test of PFBT-PCBM Pdots in 1 M KOH, 5 M MeOH and 10 µmol mL^{-1} H_2O_2 without illumination.

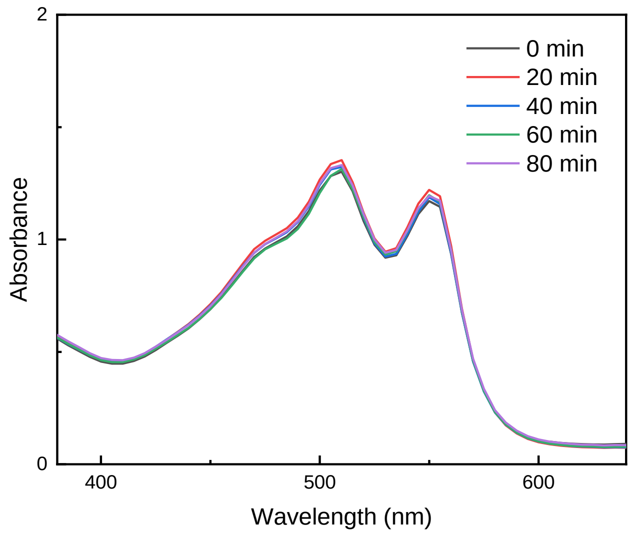

Figure S17. Concentration of H_2O_2 (initially 2 µmol mL^{-1}) measured in solution of 20 µmol mL^{-1} HCOOH, 1M KOH without adding Pdots. For each measurement, 200 µL solution was extracted and mix with 2 mL of 1M phosphate buffer solution (pH 5.4),

then, 50 µL of DPD (10 mg mL^{-1}) and 50 µL of POD (1 mg mL^{-1}) solution were added into the mixture successively.

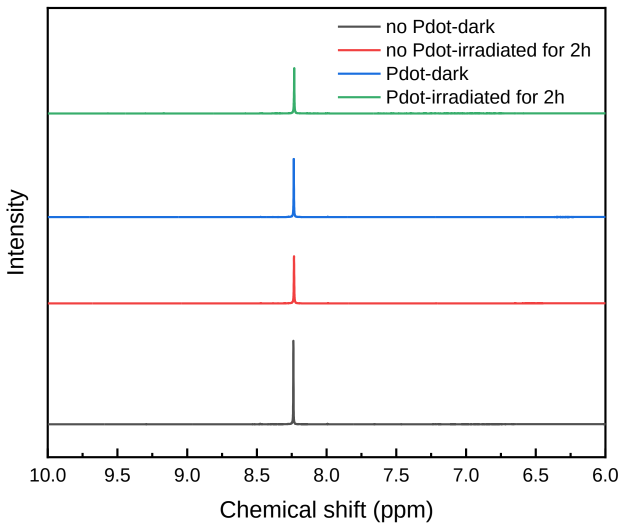

Figure S18. ^{1}H NMR spectra of 1M KOH D_2O solution containing 2 mg mL^{-1} HCHO, 20 µg mL^{-1} Pdots and 5M CH_3OD after 24 h.

Considering that depolymerization of HCHO is sluggish without heating in alkaline, both irradiated and un-irradiated samples were kept in dark for 24 h before conducting NMR measurement. This sluggish property of HCHO depolymerization is expected not to have effect during photocatalysis reaction since the Cannizzaro reaction could proceed once two HCHO molecules are generated and the self-polymerization of HCHO can be avoided. As shown in Figure S18, the signal appealed at chemical shift of 8.25 ppm is attributed to the H of $HCOO^-$ while signal for the H of HCHO at 9 ppm is not observed, which means the added HCHO was completely converted.

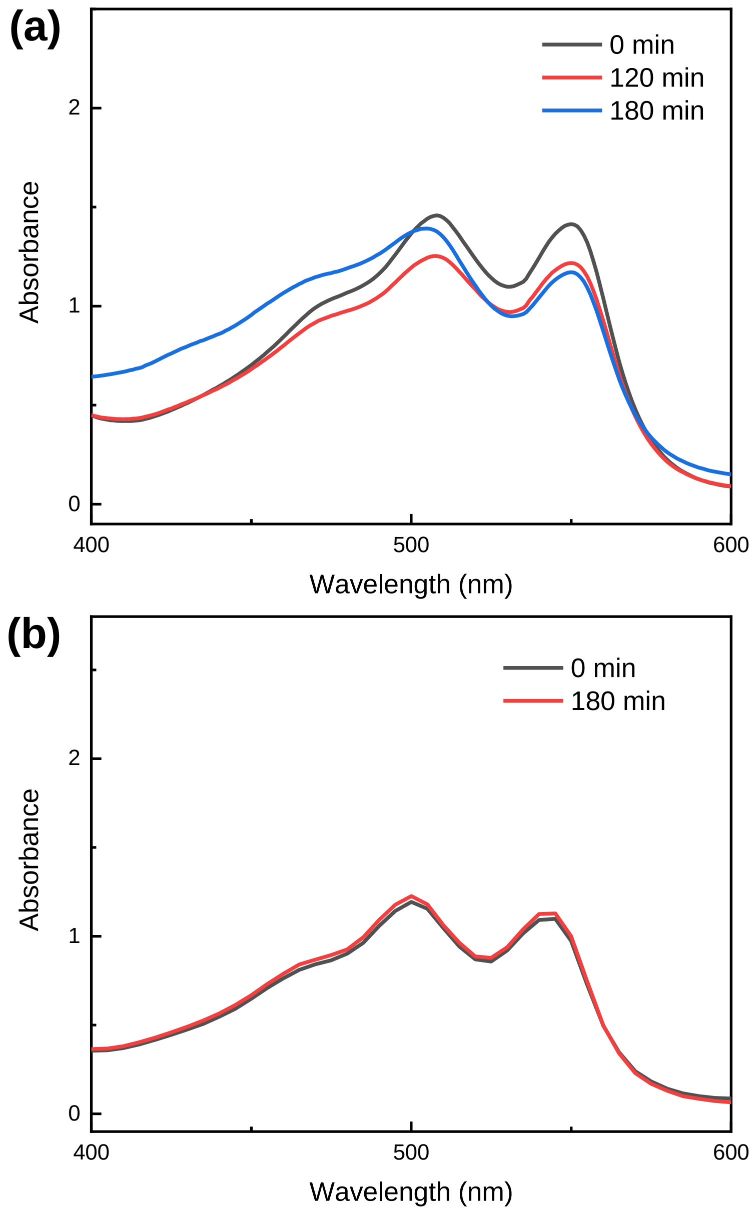

Figure S19. (a) H_2O_2 concentration in solution of 1 mM HCHO, 5 M MeOH and 1 M KOH; (b) H_2O_2 concentration in solution of 5 M MeOH and 1 M KOH. For each measurement, 200 μL solution was extracted and mix with 2 mL of 1M phosphate buffer solution (pH 5.4), then, 50 μL of DPD (10 mg mL^{-1}) and 50 μL of POD (1 mg mL^{-1}) solution were added into the mixture successively.

The results here show that HCHO could react with H_2O_2 slowly in photocatalytic system and produces formate. The stoichiometric ratio of HCHO

and H_2O_2 is calculated to be around 12:5. Therefore, the ratio of generated H_2O_2 and formate which is around 1:1 can be contributed by both Cannnizzaro reaction and the reaction between HCHO and H_2O_2.

Reference

[1] A. Liu, L. Gedda, M. Axelsson, M. Pavliuk, K. Edwards, L. Hammarstroem, H. Tian, *J. Am. Chem. Soc.* **2021**, *143*, 2875-2885.

[2] Y. Wei, J. Zhang, Q. Zheng, J. Miao, P. J. Alvarez, M. Long, *Chemosphere* **2021**, *279*, 130556.

[3] J. A. Kiernan, *Microsc. Today* **2000**, 8, 10, 12.

[4] S. Kim, G.-h. Moon, H. Kim, Y. Mun, P. Zhang, J. Lee, W. Choi, *J. Catal.* **2017**, *357*, 51-58.

[5] Y. Zheng, Y. Luo, Q. Ruan, J. Yu, X. Guo, W. Zhang, H. Xie, Z. Zhang, J. Zhao, Y. Huang, *J. Colloid Interface Sci.* **2022**, *609*, 75-85.

[6] L. Zheng, J. Zhang, Y. H. Hu, M. Long, *J. Phys. Chem. C* **2019**, *123*, 13693-13701.

[7] F. Xu, C. Lai, M. Zhang, B. Li, S. Liu, M. Chen, L. Li, Y. Xu, L. Qin, Y. Fu, X. Liu, H. Yi, X. Yang, *J. Colloid Interface Sci.* **2021**, *601*, 196-208.

[8] J. Hu, P. Zhang, T. Yang, Y. Cai, J. Qu, X. Yang, *Appl. Surf. Sci.* **2022**, *576*, 151841.

[9] L. Zhou, J. Feng, B. Qiu, Y. Zhou, J. Lei, M. Xing, L. Wang, Y. Zhou, Y. Liu, J. Zhang, *Appl. Catal., B* **2020**, *267*, 118396.

[10] Z. Wei, M. Liu, Z. Zhang, W. Yao, H. Tan, Y. Zhu, *Energy Environ. Sci.* **2018**, *11*, 2581-2589.

[11] L. Zhou, J. Feng, B. Qiu, Y. Zhou, J. Lei, M. Xing, L. Wang, Y. Zhou, Y. Liu, J. Zhang, *Appl. Catal., B* **2020**, *267*, 118396.

[12] J. Lei, B. Chen, W. Lv, L. Zhou, L. Wang, Y. Liu, J. Zhang, *ACS Sustainable Chem. Eng.* **2019**, *7*, 16467-16473.
